# Supplementary material for: Independent Evaluation of Cell Culture Systems for Hepatitis E Virus
Source: Viruses. 2022 Jun 9;14(6):1254. doi: 10.3390/v14061254 (PMC9227121; doi:10.3390/v14061254)
Supplement: Supplementary file 1 [file viruses-14-01254-s001.zip › viruses-1683582-supplementary.pdf]

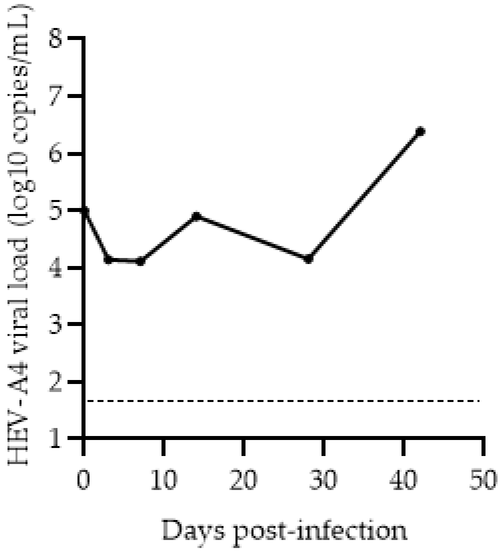

Supplementary Figure S1. Supernatant passage of hepatitis E virus (HEV) in PLC/PRF/5 cells maintained in MEM-D for HEV-A genotype 4 (HEV-A4). 0 dpi represents the viral load of the supernatant at infection. The limit of detection is denoted as the dashed line (50 copies per mL for HEV-A4).
